# Is Occupation a Risk Factor for Developing Inflammatory Bowel Disease? A Case–Control Study

**DOI:** 10.1093/crocol/otad065

**Published:** 2023-10-20

**Authors:** Violeta Mauriz-Barreiro, Alberto Ruano-Raviña, Rocío Ferreiro-Iglesias, Iria Bastón-Rey, Cristina Calviño-Suárez, Laura Nieto-García, Sol Porto-Silva, Xurxo Martínez-Seara, J Enrique Domínguez-Munoz, Manuel Barreiro-de Acosta

**Affiliations:** Gastroenterology Department, Clinical University Hospital of Ferrol, Ferrol, Spain; University of Santiago de Compostela, Santiago de Compostela, Spain; Department of Preventive Medicine and Public Health, University of Santiago de Compostela, Santiago de Compostela, Spain; CIBER de Epidemiología y Salud Pública (CIBERESP), Madrid, Spain; Clinical University Hospital of Santiago de Compostela, Santiago de Compostela, Spain; Clinical University Hospital of Santiago de Compostela, Santiago de Compostela, Spain; Clinical University Hospital of Santiago de Compostela, Santiago de Compostela, Spain; Clinical University Hospital of Santiago de Compostela, Santiago de Compostela, Spain; Clinical University Hospital of Santiago de Compostela, Santiago de Compostela, Spain; Clinical University Hospital of Santiago de Compostela, Santiago de Compostela, Spain; Clinical University Hospital of Santiago de Compostela, Santiago de Compostela, Spain; University of Santiago de Compostela, Santiago de Compostela, Spain; Clinical University Hospital of Santiago de Compostela, Santiago de Compostela, Spain

**Keywords:** epidemiology, inflammatory bowel diseases, occupation

## Abstract

**Background and aims:**

The role of occupation is uncertain in the onset of inflammatory bowel diseases. The aim of this study is to identify if there is a role of occupation in these diseases.

**Materials and methods:**

A case–control study with incident cases with inflammatory bowel diseases was designed. Cases and controls were recruited simultaneously and controls followed a sex and age frequency matching with cases. A detailed questionnaire was completed by all the participants. To analyze the results, a logistic regression was used. A subgroup analysis was performed for each inflammatory bowel disease.

**Results:**

A total of 141 patients with incident inflammatory bowel disease (80 ulcerative colitis, 55 Crohn’s disease, and 6 unclassified colitis) and 114 controls were included. There were no statistically significant differences in type of work, working hours, contact with animals, or physical activity at work between inflammatory bowel disease patients and controls. After stratifying results according to type of IBD, there were no statistically significant differences either.

**Conclusions:**

There seems to be no risk for inflammatory bowel disease onset regarding the type of work, working hours, contact with animals, or sedentariness.

## Introduction

Inflammatory bowel disease (IBD) is a group of pathologies with unknown etiology. It affects the genetically predisposed people and there are some environmental factors that have been partially identified.^1,2^ Nevertheless, little is known regarding specific risk factors related to occupation.

Some of the risk factors associated with the development of IBD are tobacco in Crohn’s disease (CD), stress, depression, anxiety, dietary habits, changes in the microbiota, gastrointestinal infections, antibiotic use, and oral contraceptives, among others. There are some protective factors too, such as smoking and appendectomy in ulcerative colitis (UC) and breastfeeding.^3–5^

The incidence of IBD varies depending on the geographical area.^6–9^ In western European countries, there is an incidence of 14 cases per 100 000 habitants per year.^10^ In the Spanish population, the study by Chaparro et al.^11^ observed an incidence of 16.2 cases of IBD per 100 000 habitants/year (8.1 cases for UC, 7.4 cases for CD, and 0.7 for unclassified colitis). For the Healthcare Area of Santiago de Compostela (where this study was carried out), the global incidence rises to 21.6 cases per year per 100 000 habitants, 6.5 for CD, and 14.2 for UC.^12^

Occupational activity has a clear impact on health. Blue-collar jobs have been defined as those entailing the highest occupational risk exposures and are also mentioned as manual workers. Classical examples of such jobs are construction workers, heavy industry occupations, or all occupations related to motor vehicles. White-collar jobs are usually defined as those with office-related work, but also with teaching or health sector. Blue-collar workers do more exhausting labor, have more physical demands, and sometimes they work outside, while white-collar workers usually work in offices doing less physical activity^13^ and are more psychologically demanding. It is also well known that sedentary behavior is associated with an increase of diabetes, cardiovascular disease, some types of cancer, and all-cause mortality.^14^ Sedentary jobs present a higher risk of obesity.^15^ Blue-collar workers are at higher risk of depression and myocardial infarction^16^ and they have high exposure to toxicants.^17^

The association between IBD and type of work has been barely studied previously,^18,19,20^ being these diseases more prevalent in white-collar employees and sedentary workers than in blue-collar ones. In the study by Furuya et al.,^21^ the risk of late-onset UC was higher in sales workers and carrying, cleaning, and packing workers, but this association was not seen in CD. The review by Leso et al.^22^ concluded that a link between occupational risk factors and IBD cannot be made yet, and more studies should be done to draw any conclusions. It is important to highlight that the available studies are scarce, somewhat outdated, and with inconclusive results, and therefore more research is warranted.

Many occupations entail the exposure of different toxic or irritant substances that may reach the bowel through different biological pathways (bloodstream or digestive tract) and act as possible triggers of these diseases. Therefore, the aim of this study is to analyze if there are differences in job classifications between recently diagnosed patients with IBD and controls, taking into account sedentariness, working hours, and occupation type.

## Materials and Methods

### Design, Subjects, and Settings

This study is based on a case–control, single-center study. All consecutive new patients diagnosed with IBD (UC, CD, and unclassified colitis) in the area of Santiago de Compostela were included. The recruitment period comprised from June 2020 to November 2022. Controls were frequency matched by age and sex with cases. IBD diagnosis was made following the diagnostic consensus guideline by the Crohn’s and Colitis Organisation and the European Society of Gastrointestinal and Abdominal Radiology.^23,24^ Controls were selected from the consults and endoscopic room of the Digestive Unit at the Clinical University Hospital of Santiago de Compostela, and these controls were subjects without IBD. To be defined as a case, a patient had to be diagnosed with IBD 3 or less months prior to the inclusion and must be over 18 years of age. Cases and controls were recruited in parallel.

The study protocol and consent forms were approved by the Santiago de Compostela-Lugo Ethics Committee (REF 2020/013). All patients and controls signed the informed consent and there were no rejections to participate.

### Information Retrieval

Data were collected by personal interviews with trained nursing staff. A detailed interview using a questionnaire developed by purpose was used. The questionnaire obtained detailed information on the lifestyle with special emphases on tobacco consumption and other habits. A specific section was dedicated to describe the occupations held (type of work, divided into white and blue collar, sedentariness at work divided into high activity, moderate activity and sedentary activity according to the classification of occupational activity categories using accelerometry,^25^ and place of work divided into indoor and outdoor), time at work, and contact with animals at work. This questionnaire also included sociodemographic information as well as the current job and past jobs (since the beginning of their career). Contact with chemicals, solvents, and dusts was taken into account, as well as sea-related occupations. These contacts were analyzed separately in a posterior analysis. Occupations were also classified as being potentially risky for IBD on the biological plausibility (ie, exposure to toxicants, chemicals, dusts, or contact with animals) on a categorical basis (yes or no).

It was analyzed if there was any relation between these variables and development of IBD.

Only for cases, to determine disease activity, the type of IBD following the Montreal classification,^26^ as well as endoscopic and clinical index (total and partial Mayo index for UC and CDAI and SES-CD for CD) and analytic parameters (CRP, fecal calprotectin, hemoglobin, ferritin, and iron) were collected.

### Statistical Analysis

Descriptive statistics is shown in percentages for categorical variables and medians and interquartile range (IQR) for continuous variables, and data were compared by χ^2^ analysis or Student’s *t*-test.

A logistic regression was carried out, taking into account age, sex, and retirement as adjustment variables. Results were presented as odds ratios with their 95% confidence intervals. A subgroup analysis was performed by each of the 3 diseases considered (CD, UC, and unclassified colitis). For the analysis of IBD disease course, only patients with IBD who were diagnosed at least 1 year since the analysis was performed were included, and flares and risk of hospitalization were analyzed and compared by type of work. Stata® 15 (StataCorp LP, College Station, TX) was used for statistical analysis.

## Results

Between June 2020 and November 2022, 141 patients with an incident diagnosis of IBD and 114 controls were included. Of the patients with IBD, 80 had UC, 55 CD, and 6 unclassified colitis. The sample size description, broken down by case and control status, is shown in Table 1. Age was very similar between cases and controls (median of 52 and 53.5, respectively) and the same occurred with sex (51.1% vs 40.4% males). In Table 2, the characteristics of patients with IBD are described.

**Table 1.** Sample size description.

	IBD patients	Controls	Total	*P*
Number (*N*)	141	114	255	
Age (median, IQR)	52 (41–58)	53.5 (39–66)	52 (40–61)	.19
Sex (*N*, %)				.22
Male	72 (51.1)	46 (40.4)	118 (46.3)	
Collar (*N*, %)				.26
Blue	54 (38.3)	37 (32.5)	91 (35.7)	
White	67 (47.5)	57 (50.0)	124 (48.6)	
NR/DK	20 (14.2)	20 (17.5)	40 (15.7)	
Activity at work (*N*, %)				
Sedentary work	62 (44.0)	47 (41.2)	109 (42.7)	
Moderate activity	36 (25.5)	32 (28.1)	68 (26.7)	.98
High activity	25 (17.7)	15 (13.2)	40 (15.7)	.60
NR/DK	18 (12.8)	20 (17.5)	38 (14.9)	
Site of work (*N*, %)				.06
Outside	35 (24.8)	20 (17.5)	55 (21.6)	
Inside	103 (73.0)	94 (82.5)	197 (77.3)	
NR/DK	3 (2.1)	0	3 (1.2)	
Animal contact (*N*, %)				.74
Yes	18 (12.8)	12 (10.5)	30 (11.8)	
No	106 (75.2)	82 (72.0)	188 (73.7)	
NR/DK	17 (12.1)	20 (17.5)	37 (14.5)	

Abbreviations: IBD, inflammatory bowel disease; IQR, interquartile range; NR/DK, No response/don’t know.

**Table 2.** Description of patients with inflammatory bowel disease.

	Ulcerative colitis	Crohn’s disease	Unclassified colitis
*N* (number)	80	55	6
Age (median, IQR)	52.5 (43–59)	52 (37–59)	46.5 (34–52)
Sex (*N*, %)			
Male	43 (53.8)	27 (49.1)	2 (33.3)
Female	37 (46.3)	28 (50.9)	4 (66.7)
Classification (*N*, %)	Proctitis: 30 (37.5)	L1: 35 (63.6)	
	Left-side colitis: 29 (36.3)	L2: 9 (16.4)	
	Pancolitis: 21 (26.3)	L3: 9 (16.4)	
		L4: 2 (3.6)	
		B1: 30 (54.5)	
		B2: 18 (32.7)	
		B3: 7 (12.7)	
Disease activity (*N*, %)			
Mild	31 (38.8)	16 (29.1)	
Moderate	47 (58.8)	29 (52.7)	
Severe	2 (2.5)	10 (18.2)	
Smoking habit			
Never smokers	36 (45.0)	15 (27.3)	3 (50.0)
Current smokers	2 (2.0)	20 (36.4)	1 (16.7)
Ex-smokers	42 (52.5)	20 (36.4)	2 (33.3)

Abbreviation: IQR, interquartile range.

Of the 114 controls, 37 (32.5%) were blue-collar workers and 57 (50%) were white-collar workers. Of the IBD patients, 54 (38.3%) were blue-collar and 67 (47.5%) white-collar workers. The majority of the workers had an indoor job (82.5% for controls and 73.0% of IBD patients) without contact with animals (72% with no contact in the control group and 75.2% in the IBD patients). Type of work, divided into white collar and blue collar, was compared between cases and controls, adjusting by age, sex, and retirement status. There were no statistically significant differences between groups (*P* > .05).

There were no statistically significant differences between patients with IBD and controls in relation to contact with animals at work, nor in outdoor workers neither indoor ones (*P* > .05). Contact with solvents, dusts, and chemicals was not associated with the development of IBD (*P* > .05), nor working in sea-related activities (*P* > .05).

Taking into account the physical activity at work, most cases and controls had a sedentary work (41.2% in the group of controls and 44.0% in IBD patients) compared with moderate activity (28.1% and 25.5%, respectively) and high activity (13.2% for controls and 17.7% for cases). Physical activity at work, divided into sedentary work, moderate activity, and high activity, was not associated with the appearance of IBD (*P* > .05). Results are shown in Table 3. Following stratification according to type of IBD, there were no statistically significant differences.

**Table 3:** Results of the logistic regressions.

	IBD patients	Controls	OR (95% CI)
Type of work (*N*, %)			
White collar	67 (47.5)	57 (50.0)	1
Blue collar	54 (38.3)	37 (32.5)	1.3 (0.7–2.3)
NR/DK	20 (14.2)	20 (17.5)	
Activity at work (*N*, %)			
Sedentary work	62 (44.0)	47 (41.2)	1
Moderate activity	36 (25.5)	32 (28.1)	0.9 (0.5–1.7)
High activity	25 (17.7)	15 (13.2)	1.3 (0.6–2.8)
NR/DK	18 (12.8)	20 (17.5)	
Site of work (*N*, %)			
Inside	103 (73.0)	94 (82.5)	1
Outside	35 (24.8)	20 (17.5)	1.7 (0.9–3.4)
NR/DK	3 (2.1)	0	
Animal contact (*N*, %)			
No	106 (75.2)	82 (72.0)	1
Yes	18 (12.8)	12 (10.5)	1.3 (0.6–2.6)
NR/DK	17 (12.1)	20 (17.5)	

Abbreviations: CI, confidence interval; IBD, inflammatory bowel disease; NR/DK, no response/don’t know; OR, odds ratio.

## Discussion

IBD is an illness with increasing incidence and mostly unknown etiology and in which specific risk factors are not well known. In this study, no differences between recently diagnosed IBD patients and controls were observed taking into account the type of work, activity at work or contact with animals, dust exposure, chemicals, or specific risk professions.

It is well known that occupational activity can affect health. Depending on the type of job, people are prone to different illnesses, such as diabetes, cardiovascular disease, and some types of cancer in sedentary workers^14^ or more risk of obesity in sedentary jobs and in longer working hours.^15^

Regarding IBD, the association with type of work has been studied previously,^18,19^ being more prevalent in white-collar employees and sedentary workers than in blue-collar ones. However, the number of studies analyzing job and IBD are scarce and they do not allow reaching a conclusion. Furthermore, most of these studies were carried out more than 25 years ago, before the start of biologic treatments and when working conditions were different from now. These studies are not prospective, in contrast with this one in which all consecutive new patients were included.

Current evidence on how work exposure can influence the development of IBD or its progress is not well established. Leso et al. made a review of the literature,^22^ but it was inconclusive. There is some information about exposure to metals and development of IBD. For example, aluminum can induce a T-helper (Th-1) inflammatory response that can lead to the development of IBD in animal models. Perfluorooctanoic acid (PFOA) was also associated with CD incidence. Industrialized environments have more incidence of IBD,^9^ so industrial activity could have a role in its development. Contact with animals or its microorganisms could have a role in developing IBD also, for example, contact with *Mycobacterium avium* subspecies *Paratuberculosis* (MAP), although controversial, was associated with CD. Contact with yeast and hypersensibility to *Saccharomyces cerevisiae* was associated with CD, with bakers having a possible higher risk of developing IBD. Working outside could be a protective risk factor in the development of IBD, because outside workers have more sun exposure, and this exposure is suggested as a protective factor of IBD.^9^ Activity at work was related to IBD too. In a study by Sonnenberg et al.,^19^ an inverse correlation was found between physical activity at work and IBD development or its mortality. It was postulated that sedentariness was associated with augmented intestinal transit time, with more time for immunologic reactions with the antigens.^20^

This study has some limitations. The sample size is somewhat low, particularly if we consider the separate analysis between CD and UC. Nevertheless, we are of the opinion that having included all incident cases occurring in a geographical area may partly overcome this limitation. Furthermore, the number of controls is quite high. A further limitation is the heterogeneity in the occupational history, which means that both cases and controls can have risky or nonrisky occupations during their lifetime. Nevertheless, the fact of having a comparison group allows us to test this hypothesis since we should expect that cases should have more occupations posing a suspicion of involvement on the onset of the disease compared to controls. It is true that the type of occupation has a huge variability worldwide, and in this study, there is a high proportion of patients with white-collar jobs. It could be that in non-developed countries, this could not be representative. Furthermore, part of this study was carried out during the COVID pandemic, and, at least during a few months, some patients could have changed their work activity. Another limitation is the correlation between occupation and other potential environmental risk factors of IBD such as smoking, alcohol intake, and others. It is well known that particularly blue-collar workers smoke and drink more frequently. Due to the limited sample size of this study, a further analysis on this aspect was not possible. Another limitation is that the questionnaire has not been formally validated, but previous versions have been used to assess the role of occupation in other diseases such as lung cancer (which has a clear relationship with occupation). One last limitation is that no psychological evaluation was performed on the patients of the study, which is a confounding factor because symptoms can be exacerbated by stress and not only by disease activity. But objective parameters such as analytic values were taken into account, minimizing the weight of clinical parameters alone.

The strength of this study is that, due to the universal coverage of the Spanish health system, IBD cases are representative of those in the community, reducing the possibility of a selection bias. Furthermore, in this study, a questionnaire specifying occupation was filled out in a personal interview carried out by trained personnel. Another strength is that only incident cases were included.

To conclude, in this study, there is no association between type of work, working hours, contact with animals, or sedentariness and developing of IBD.

## Data Availability

The data that support this article are available from the corresponding author on reasonable request.
